# Learning from parental experience in a neonatal surgical unit: a qualitative service evaluation

**DOI:** 10.1136/wjps-2023-000596

**Published:** 2023-07-10

**Authors:** Anna Littlejohns, Emile Crouzen, Rebecca Mernenko, Fiona Metcalfe, Waaka Moni-Nwinia, Hemma Chauhan, Bethan Johnson, Douglas McConachie, Elizabeth Lawson, Victoria Tricklebank, John G McElwaine, Gurdeep S Sagoo, Liz McKechnie, Gary Latchford, Jonathan Sutcliffe

**Affiliations:** 1 Anaesthesia and Critical Care, Leeds Teaching Hospitals NHS Trust, Leeds, UK; 2 Paediatric Surgery, Leeds Children's Hospital, Leeds, UK; 3 Leeds Centre for Newborn Care, Leeds Children's Hospital, Leeds, UK; 4 Faculty of Medicine and Health, University of Leeds, Leeds, UK; 5 Academic Unit of Health Economics, University of Leeds, Leeds, UK; 6 Institute of Health Sciences, University of Leeds Leeds, Leeds, UK; 7 St James's University Hospital Department of Clinical and Health Psychology, Leeds, West Yorkshire, UK

**Keywords:** neonatology, pediatrics, qualitative research, intensive care, neonatal, health services research

## Abstract

**Objectives:**

Patient experience is directly related to health outcomes, and parental experience can be used as a proxy for this in neonatal care. This project was designed to assess parental experience of neonatal surgical care to inform future service developments and improve the care we provide.

**Methods:**

This was a qualitative study using rapid qualitative analysis. The study was carried out in a large neonatal surgical intensive care unit in the UK. Parents of infants treated by the neonatal surgical team between March 2020 and February 2021, during the COVID-19 pandemic were included. Purposive sampling was used to ensure that a representative range of parents were interviewed. A semistructured interview was created and tested in a previous phase of work. This questionnaire was used to ask parents open questions about different aspects of their infants’ healthcare journey from the antenatal phase through to discharge from the neonatal unit (NUU).

**Results:**

Rapid qualitative analysis was employed, and parental experiences were grouped into five main categories: before admission to the NNU, initial admission to NNU, information and support, COVID-19 and discharge. Within these five groups, we highlighted positive experiences to be fed back to the healthcare teams to reinforce good practice, areas that warranted improvement and suggestions for service development.

**Conclusions:**

The wealth of data generated from the interviews has been summarized and shared with healthcare teams who are putting the service improvement suggestions into practice. The tool is available for services that wish to measure parental experience.

**WHAT IS ALREADY KNOWN ON THIS TOPIC**

- Parental experience is hugely important in the neonatal surgical setting, particularly in the context of family integrated care. However, formal assessment of this is lacking.

**WHAT THIS STUDY ADDS**

- This is the first use of our recently-developed interview tool to gain an in-depth understanding of parental experience on the neonatal unit from admission to discharge.

**HOW THIS STUDY MIGHT AFFECT RESEARCH, PRACTICE OR POLICY**

- Using the observations of a representative range of families, key areas of good practice and areas for improvement have been identified, leading to actionable suggestions for service improvements. Workstreams have been created to implement these improvements. Since these improvements were based partly on the perspective of ‘difficult to access’ groups, they may be more likely to successfully improve parental experience for the whole cohort of families we look after. In turn this is likely to reduce health inequality, be more effective and reduce wasted effort. While our findings can be adapted to assess and improve neonatal surgical care in other centers, the tool may be applied to other settings to define and address the needs of patients, service providers and other key stakeholders.

## Introduction

Positive patient experience is associated with improved health outcomes^1^ as well as being intrinsic to the delivery of humane care. Within the neonatal setting, parental experience can be used as a proxy for patient experience.^2^ The Neonatal Critical Care Review emphasizes the need for enhancing the family experience.^3^ Nevertheless, the ‘Getting It Right First Time’ (GIRFT) report for pediatric general surgery and urology in England and Wales acknowledges that ‘the method of collecting patient experience data is lacking for pediatric surgery’,^4^ and the National Institute of Health and Care Excellence (NICE) guideline ‘Babies children and young people’s experience of healthcare’ notes that since particular groups may be less likely to provide feedback, their views should be actively sought.^5^

Family integrated care (FICare) is an important component of modern neonatal practice. It establishes parents as partners in care by providing education and psychosocial support to enable them to gain confidence and become their infant’s main caregiver. FICare improves health outcomes, including parental experience,^6 7^ and our unit introduced a model of FICare in 2017.

In March 2020, the SARS-CoV-2 (COVID-19) pandemic abruptly changed the delivery of healthcare. A large-scale review demonstrated that the restrictions significantly negatively affected the care provided for neonates and led to a poor experience for parents, the wider family and healthcare professionals.^8^ The authors highlight how bonding and developmental care practices suffered and articulate the unique characteristics of high-quality neonatal care and the extreme vulnerability of many neonatal patients.^9^ A key message was that an in-depth understanding of the unintended consequences that COVID-19 has had in a neonatal setting was needed. There was also a need to create tools and guidelines to be able to adapt to any ongoing or future changes.^8^

Our project was designed in the early stages of the pandemic to capture how parental experience of neonatal surgical care had been affected and to inform future service developments.

## Methodology

### Participants

Participants in the study include parents of infants treated by the neonatal surgical team during the COVID-19 pandemic, between March 2020 and February 2021 were reruited in this study.

### Design

A semistructured interview was developed and tested (online supplemental table 1). Key characteristics of patients and families had been defined previously to guide purposive sampling.^10^ Information about the project was advertised using posters, social media and through the neonatal unit (NNU) staff. Families interested in participating were provided with written and verbal information available in a range of languages. Each interview was conducted by two members of the project team. Audio recordings were taken to enable accurate transcription. One interviewer transcribed the interviews verbatim. The interview team comprised 10 members and included nurses, a nurse manager, a trainee advanced clinical practitioner, and trainee and consultant surgeons and neonatologists. All were trained and coached in interview techniques and qualitative analysis by a clinical psychologist.

10.1136/wjps-2023-000596.supp1 Supplementary data

The project was delivered without funding except for translation services, supported by our pediatric surgery department.

### Patient and public involvement

Parents are used as proxies for the patients in the neonatal setting. Parents have been integral to this work from design to completion. Parents were key stakeholders involved in the initial design, creation and cognitive testing of the interview tool. A different group of parents were the participants who were interviewed to generate our results and they have given suggestions for service improvements going forwards.

### Analysis

Qualitative analysis is typically complex, time-consuming and arguably unsuitable in situations where information is sought quickly, such as during a health crisis. Thus, the project was informed by the ‘rapid assessment process’^11^ and particularly ‘rapid qualitative analysis’, which was adapted for this study.^12 13^ Traditional qualitative methods involve detailed and time-consuming analysis of transcriptions which the researcher reads and re-reads repeatedly while they identify emergent themes and their relationship to each other, attempting to capture the experiences of participants. In contrast, rapid qualitative analysis is a form of ‘top-down’ analysis where many of the parameters are defined from the start, it is designed to answer specific questions about the service rather than produce a theoretically driven account of patient experience.

The interview questions provided the framework for the analysis. Each question was summarized using a neutral domain name, for example, ‘preparation for leaving the unit’. A summary template was written listing all questions and domains, with columns for participant responses and quotations. An example of a summary template used in the analysis (online supplemental table 2). Any responses that did not fit existing domains were added to a new category. The summary template was piloted by the six members of the analysis team on one interview to test suitability. Minor changes were made to domains and an extra column detailing possible service implications was added. When consistency was established, transcripts were divided between the team for analysis, with each analyzed independently by two team members who agreed on a final summary for each participant. All summaries were combined to produce an initial matrix for all participants. The matrix was then divided between five members of the team who produced summary matrices for five aspects of the patient journey: before admission to the NNU, initial admission to the NNU, information and support, COVID-19 and discharge. A final summary of parental themes and service implications was created.

10.1136/wjps-2023-000596.supp2 Supplementary data

## Results

Twenty-four participants were recruited.Each of the characteristics deemed important by the stakeholder analysis in the phase I work were represented by at least one of the families recruited (table 1).^10^ A total of 18 interviews were carried out, 6 interviews with parents together and 12 interviews with parents separately. A mixture of virtual and face-to-face interviews were undertaken as determined by participant preference. Interviews were typically between 30 min and 90 min. Descriptive results are presented further. The summary results tables (online supplemental tables 3–7).

10.1136/wjps-2023-000596.supp3 Supplementary data

**Table 1:** Participant characteristics

Participant characteristics: infant			Participant characteristics: parent		
Presentation (%)	Acute	96	Depravation index (1–10) (%)	1–5	54
	Elective	4		>5	42
				Unknown	4
Number of siblings (%)	0	38	Mother’s age (years) (%)	<20	8
	1	33		20–25	8
	2	4		26–30	21
	3+	21		31–40	33
	Unknown	4		40+	17
Multiple morbidity (%)	Yes	71	Single parent (%)	Yes	13
	No	29		No	83
				Unknown	4
Antenatal diagnosis (%)	Yes	25	Marital status (%)	Single	21
	No	75		Cohabiting	38
				Married	33
				Separated/divorced	0
				Unknown	8
Length of stay (days) (%)	1–14	8	Ethnicity (%)	White British	67
	15–31	42		Other European	17
	>31	50		African	4
				Asian	4
				Unknown	8
Care at another hospital (%)	Yes	79	First language (%)	English	76
	No	21		Other European	17
				Other	8
Care in another department within trust (%)	Yes	25	Highest education level (%)	None	4%
	No	75		Some high school	13
				High school	17
				College	17
				Bachelor’s degree	13
				Master’s degree	8
				Unknown	29
Highest level of care (%)	Ward	13	Internet at home (%)	Yes	58
	HDU	8		No	13
	NICU/PICU	79		Unknown	29
Specialty (%)	Upper GI/thoracic	12.5	Disability (%)	Yes	0
	Lower GI	87.5		No	67
				Unknown	33
Gestation at birth (weeks) (%)	24–27	33	Travel time to hospital (min) (%)	<20	21
	28–31	4		20–39	4
	32–35	21		40–59	38
	36+	42		60+	8
				Unknown	33
Gestation at presentation (weeks) (%)	24–27	4	IVF (%)	Yes	21
	28–31	21		No	60
	32–35	21		Unknown	29
	36+	54			
Current gestation (weeks) (%)	24–27	0	Multiple pregnancy (%)	Yes	8
	28–31	4		No	88
	32–35	4		Unknown	4
	36+	83			
	Unknown	8			

GI, gastrointestinal; HDU, high-dependency unit; IVF, In vitro fertilization; NICU, neonatal intensive care unit; PICU, paediatric intensive care unit.

### Before admission to NNU

There was sometimes confusion among the parents who received a diagnosis postnatally as to why the diagnosis had been made antenatally, even for conditions not typically identified antenatally. A sense that a diagnosis may have been missed lowered confidence in the clinical team:

*’We thought if anything would have been wrong, it would have shown on the amniocentesis.’*

The lack of diagnosis led to an inability to prepare for admission:

*’[It was] overwhelming at first…felt hysterical at first but calmed down.’*

When a diagnosis had been identified antenatally, both partners being able to access counseling and appointments together was important:

*’It is really upsetting to talk about it to be honest. I don’t think I have processed it myself yet.’*

*’I had to do all the scans on my own which I found really upsetting.’*

### During admission to NNU

The initial transfer to the NNU was identified as a particularly stressful event:

*’The transfer from one unit to another unit was the worst part of it all. It took a long time for the transfer to happen.’*

*‘I think a midwife should have taken me to neonatal, that would have been very helpful; they could have then said ‘this is [baby]’s mum, could someone please show her around.’*

A recurrent theme was the importance of personalizing care:

*‘Young mums need a bit more support and a bit more explanation.’*

*‘I had had a C-section on a different ward and were unable to go anywhere, while [baby] was going to theatre, I could not come to [baby].’*

Staff kindness was easily recognizable, and the importance of the broader team on health outcomes was emphasized:

*‘To say it was a really rubbish situation it was really lovely, they were all great, understanding.’*

Comments on accommodation and non-clinical areas provided actionable information for service planning for established and potential new build:

*‘It’s not a hotel but everything is perfect still.’*

*’Got permit for free parking but there was still not always a parking space. Drove around a lot.’*

Comments on the clinical areas highlighted the need for increased privacy and natural light:

*’…was ridiculous to deprive the babies of this much light.’*

The financial burden was acknowledged:

*’It is extremely expensive to have a baby admitted to [hospital] due to the costs around it.’*

The availability of a kitchen to help decrease costs for families, lockable cupboards, free coffee and tea, reliable free Wi-Fi and a video link to access the ward when needed were all regularly raised and described as ‘a lifesaver’. These interventions are relatively inexpensive ways to improve experience.

### Information and support

Communication was a key theme, and overall, information was felt to be clearly presented and regularly updated:

*’The amount of information about the baby was enormous; thanks to the quality of the team, I understand everything.’*

Diagrams were felt to be particularly useful, for example, to help visualize aspects of anatomy. Some families felt that staff were not always available, for example, at weekends. Understanding how to access the team was not always clear, especially early in the stay: what to do if the ward round did not enter their cubicle, for example.

Honesty was highly valued, including for bad news:

*’I didn’t feel that they were keeping any secrets. That’s sometimes what you worry about, is there something they are not telling me.’*

However, when there had been a loss of trust, this had important consequences:

*‘There was an incident when [baby] got an abscess from a cannula that was inserted. We did not know how honest they were about this. … but it felt like they were trying to shove it under the carpet in fear of us complaining.’*

Continuity of care across staff groups appeared important:

*‘It is almost like you have the same nurse for the whole time really that you are in hospital because they all know what is going on and it is great.’*

Most knew and appreciated the offer of talking to a counsellor, but the team, particularly the nurses, were also an important source of support. Information about the experience of bereavement was striking; small things mattered and had a lasting impact:

*‘The stuff they did before [X] died and afterwards, it was never too much. Like we left some of his clothes here and they posted them out to us… I don’t think I could have been as strong as I was if they weren’t as strong as they were as well.’*

Participants with limited English appreciated the efforts to find interpreters but expressed a preference for quicker solutions:

*’I would be happy with Google translate sometimes.’*

### COVID-19 impact

Parents generally felt they and their baby were safe although desperate to get them home to their ‘little bubble’. They worried they were potentially vulnerable:

*’COVID was always at the back of the mind.’*

Strict implementation of infection prevention guidance was seen as reassuring. There was frustration over discrepancy between testing for visitors and staff:

*‘[We] Don’t really believe in COVID but accept restrictions.’*

*’It was just strange that the medical staff were not being swabbed.’*

Parents were able to clearly articulate the impact of restrictions:

*’It must be horrible for babies to just feel rubber and plastic all the time.’*

*‘The only people who were touching her were medical staff. For 5 weeks we did not hold our baby, which made the bond we initially had disappeared. The only people touching her were medical staff for interventions.’*

### Discharge

Parents generally felt well prepared for discharge; they noted help with paperwork and good follow-up from the surgeon and the team. Support from clinical nurse specialists, the community team and outreach nurses was important. This included emotional support, noted particularly by one parent who sadly lost their baby:

*’There is nothing I can imagine that could have helped us more.’*

The distress generated when discharge was delayed was striking. Provision of ‘goal-based’ criteria for discharge might allow less focus on a specific time:

*’It is a strange feeling to have your baby at home after long invasive support, which is why still having access in the community is essential.’*

With the benefit of hindsight, parents often acknowledged that they were not as well prepared psychologically as they thought, and they have become hypervigilant:

*‘A bit of a shock to the system, but nothing more could’ve been done.’*

*’I just have to be more careful and really watch my baby for any signs.’*

### Service improvements

The insights into parental experience have led to local service improvements and influenced projects already underway. Various workstreams were created to formulate meaningful responses to the parents’ concerns, thereby developing our service and improving the care we provide. A summary of the resultant improvements is shown in table 2. A comprehensive list of the improvements made and further improvements planned can be found in online supplemental tables 3–7.

**Table 2:** Examples of service improvements

Aspect of patient journey	Examples of service improvements informed or instigated
Before admission to NNU	Monthly fetal medicine multidisciplinary team meetings and 6 monthly reviews of fetal medicine clinic. Video tour of NNU now available prior to admission. Midwives now bring mothers to the NNU on their first visit, and the family care team provides orientation to the unit.
During admission to NNU	Psychology service now in place. Parents are actively encouraged to attended ward round. Their attendance is recorded and audited across the service. Badgernet video diaries used frequently to connect families with their baby when not on the unit. New screens purchased to help provide more privacy. Parking permits available for families, some designated parking spaces protected for neonatal family use.
Information and support	Joint neonatal and surgical ward rounds from Monday to Friday and weekly multiprofessional team meetings to have holistic oversight of progress and ongoing care planning. Every patient has a named neonatologist and named surgeon, with this displayed by the bedside. Poster with details and picture of staff uniforms and job roles to help families understand different staff roles. Hospital chaplaincy team starting to facilitate weekly coffee morning for families. Our new NNU podcast ‘Unexpected Beginnings: The Neonatal Unit’. This is hosted by veteran neonatal parents and runs through key aspects of being a parent on the NNU to provide support for other parents.
COVID-19 impact	Regular communication and letters given to all parents in relation to any infection prevention control issues. Parental feedback on COVID-19 concerns disseminated to all teams involved in neonatal care.
Discharge	Implementation of criteria-led discharge to help manage parental expectations and reduce delays on day of discharge. Multidisciplinary discharge meetings arranged for more complex infants, district general hospital teams invited virtually if there is a surgical neonate returned to local center. Weekly ‘discharge huddle’ to discuss patient flow, outstanding tasks and any family needs. Extra basic life support training sessions for staff so more staff is able to support parental training and reduce delays on discharge.

NNU, neonatal unit.

## Discussion

The value of measuring parental experience in the neonatal setting is well recognized. Despite this, NICE and GIRFT have highlighted the lack of mechanism for collecting these data and make a clear recommendation that this should be addressed.^4 5^

The impact of COVID-19 on neonatal care is emerging in the literature. Given the importance of FICare, it is understandable how visiting restrictions have been particularly detrimental in this setting. Work from across the globe has demonstrated how restrictions have negatively impacted parental well-being, increased parental concerns about bonding and childhood development, and reduced parental confidence in caring for their infant.^9 14–16^ Our study adds support to these findings and offers further insight from groups that are representative of the range of families we look after, including families traditionally seen as ‘difficult to access’. We have interviewed parents with a range of characteristics (eg, deprivation index, health literacy, first language, single-parent and two-parent families, ages, and distance travelled) whose infants themselves had a range of characteristics (eg, gestational age, disease complexity, and length of stay). We believe that this has made the information obtained more likely to reflect real-world experience and make the attempt to improve service delivery more likely to be effective.

It is valuable to have the perspective of families who have observed the service closely for many hours. Many of the issues raised have simple solutions, and we are collaborating with healthcare teams to develop and implement change. A number of the areas for improvement can be addressed by building on the ‘culture and values’ already in place. Other recommendations, such as the importance of kitchens, can be used to inform the plans for our new children’s hospital, currently under development. The insights into the direct and indirect impact of COVID-19 will inform preparation for potential future challenges. However, despite the context of the pandemic, most of the themes were not related exclusively to COVID-19. Therefore, we believe our results can give insight more broadly into the general experience of parents on the surgical NNU.

The amount of information obtained was extensive. This is hard to present academically and challenging to manage clinically. The main value of this project is taking these results forward so they can lead to service development. Several workstreams have been created, for example, the antenatal workstream, with input from relevant healthcare groups to prioritize the themes and actionable improvements.

From the beginning of this project, we understood the potential for scalability to other clinical pathways. Other aspects of care (clinical outcomes, process, and resource use) might usefully be considered by assessing cognitive diversity, and the methodology used here may be applicable. A recent study into crisis leadership in the health service in Slovakia during the pandemic found that having a detailed understanding of the perspective of representative stakeholders allowed cognitive diversity and was a source of trust, satisfaction, and engagement in medical teams and helped inform rapid decision making.^17^ As in many fora outside medicine, it seems likely that capturing a variety of perspectives, and understanding the different needs and thought processes of a curated range of people will usefully add rigour to how services are designed.

We hope our interview tool and findings can be of use in other settings. While some observations may be specific to our center, others may be directly applicable elsewhere. The interview tool, however, is something that can be used widely.

## Data Availability

Data are available upon reasonable request.
