# Supplementary material for: Learning from parental experience in a neonatal surgical unit: a qualitative service evaluation
Source: World J Pediatr Surg. 2023 Jul 10;6(3):e000596. doi: 10.1136/wjps-2023-000596 (PMC10335473; doi:10.1136/wjps-2023-000596)
Supplement: Supplementary data [file wjps-2023-000596supp001.pdf]

Date and Time of Interview

Consent recorded? Y/N

Purpose of the interview and what we want them to do

Set out the main areas there will be questions so each get enough focus (e.g, before admission and early on, then during the stay, then communication, COVID-19, and thoughts around leaving the unit)

Thinking about when you were pregnant and when your baby was first born

1- What were your main worries about the thought of coming into hospital?
    a. Were you worried about the pandemic, or did the pandemic have any other affect?
2- Did you know what to expect for you and your baby's care once your baby was born?
    a. Did you think that the pandemic might have an effect?
3- Did you know who the team looking after your baby were?
    a. Did you know which consultant neonatologist and which consultant surgeon was in charge of your baby's care, and how the 2 link?
4- Were you made to feel welcomed by the team looking after your baby?
    a. Did you feel that they were approachable?

Thinking about the neonatal unit

1. What are your thoughts on the room(s) where your baby was?
2. What do you think about your privacy while on the ward?
    a. Do you think the pandemic affected your privacy?
3. Do you have any other comments regarding practical things that made a difference to you? For example, the parents' accommodation, refreshment areas, anything to do with your ability to travel to and from the hospital to visit or parking?
    a. Did you notice any effects on these that might have been caused by the pandemic?
4. Is there anything else about practicalities of being on a neonatal unit we should have asked you?

Considering the information and support you received

1. For your personal circumstance did you need additional help (for example an interpreter) and was this provided for you?
2. Did you understand your baby's diagnosis and treatment as much as you would have liked?
    a. Were you able to talk to nursing staff and the doctors as much as you wanted?
3. How do you feel about the amount of information you were given?
    a. Too much? Too little?
4. Were you ever given conflicting information?
5. Were you aware of how to access emotional and practical support for you before, during or after your baby's admission? Was there anyone who was a 'constant' person all the way through?

6. Is there anything you would have liked to have known that wasn't in the information given to you?
7. Do you think the pandemic affected the communication you received?
8. Were the rules about COVID-19 easy to understand?
    a. Were the rules always followed the same way by different staff members or families? Did you notice differences between rooms? What happened when rules weren't followed?
9. Do you recall the rules changing?
    a. If so, how soon did you get to know?
10. Did you feel confident that information about your baby's care was shared well between the members of the team, including nurses, doctors and everyone else in the team?

Thinking about COVID-19

1. Did the pandemic change how safe you felt for you and your baby?
2. Do you think that the pandemic and its regulations influence how you and anyone else important for your baby (for example your partner) were able to bond with your baby?
3. Do you have other children? Has having your baby in hospital affected them?
    a. Do you think that the pandemic affected this?
4. Are there ways in which you think the pandemic might have affected the way that staff cared for your baby?
5. Are there any other sources of stress that we have not asked about?

Leaving the unit

1. When you leave the neonatal unit, do you know if it will be to another ward, or to go home? How prepared do you/did you feel for discharge from the neonatal unit?
2. Did someone make you aware of the support that is available to you for when you do get home, for example, coming to A&E, seeing your GP, support from family?
3. Do you have any particular concerns for your baby once they are at home, having had surgery?
4. Are there any additional concerns or practical problems caused by the pandemic?

Time Interview Finished

Supplementary Table 1. Interview Questions
