# Supplementary material for: Learning from parental experience in a neonatal surgical unit: a qualitative service evaluation
Source: World J Pediatr Surg. 2023 Jul 10;6(3):e000596. doi: 10.1136/wjps-2023-000596 (PMC10335473; doi:10.1136/wjps-2023-000596)
Supplement: Supplementary data [file wjps-2023-000596supp002.pdf]

Question	Domain
**Thinking about when you were pregnant and when your baby was first born**	
What were your main worries about the thought of coming into hospital?	General anticipatory worries (including effect of COVID-19)
Were you worried about the pandemic, or did the pandemic have any other affect?	
Did you know what to expect for you and your baby's care once your baby was born?	Expectations of newborn care (including effect of COVID-19)
Did you think that the pandemic might have an effect?	
Did you know who the team looking after your baby were?	Knowledge of team
Did you know which consultant neonatologist and which consultant surgeon was in charge of your baby's care, and how the 2 link?	
Were you made to feel welcomed by the team looking after your baby?	Feeling welcomed
Did you feel that they were approachable?	
**Thinking about the neonatal unit**	
What are your thoughts on the room(s) where your baby was?	Parental view: room
What do you think about your privacy while on the ward?	Parental view: privacy (including effect of COVID-19)
Do you think the pandemic affected your privacy?	
Do you have any other comments regarding practical things that made a difference to you? For example, the parents' accommodation, refreshment areas,	Parental view: practicalities (including effect of COVID-19)

anything to do with your ability to travel to and from the hospital to visit or parking?	
Did you notice any effects on these that might have been caused by the pandemic?	
Is there anything else about practicalities of being on a neonatal unit we should have asked you?	
**Considering the information and support you received**	
For your personal circumstance did you need additional help (for example an interpreter) and was this provided for you?	Presence of additional help (if needed)
Did you understand your baby's diagnosis and treatment as much as you would have liked?	Understanding /ability to talk to team
Were you able to talk to nursing staff and the doctors as much as you wanted?	
How do you feel about the amount of information you were given?	Information amount
Too much? Too little?	
Were you ever given conflicting information?	Presence of conflicting information
Were you aware of how to access emotional and practical support for you before, during or after your baby's admission? Was there anyone who was a 'constant' person all the way through?	Access to emotional/practical support:<br>Before – During – After; consistency
Is there anything you would have liked to have known that wasn't in the information given to you?	Unmet information needs
Do you think the pandemic affected the communication you received?	Information about COVID-19
Were the rules about COVID-19 easy to understand?	Understanding of COVID-19 rules (differences/changes/consistency?)
Were the rules always followed the same way by different staff members or families? Did you notice differences between rooms?	

What happened when rules weren't followed?	
Do you recall the rules changing?	
If so, how soon did you get to know?	
Did you feel confident that information about your baby's care was shared well between the members of the team, including nurses, doctors and everyone else in the team?	Parental view: information was shared between team
**Thinking about COVID-19**	
Did the pandemic changed how safe you felt for you and your baby?	Feeling safe
Do you think that the pandemic and its regulations influence how you and anyone else important for your baby (for example your partner) were able to bond with your baby?	Impact on bonding with baby
Do you think that the pandemic affected this?	
Do you have other children? Has having your baby in hospital affected them?	Impact on siblings
Do you think that the pandemic affected this?	
Are there ways in which you think the pandemic might have affected the way that staff cared for your baby?	Parental view: impact on staff
Are there any other sources of stress that we have not asked about?	Other sources of stress
**Leaving the unit**	
When you leave the neonatal unit, do you know if it will be to another ward, or to go home? How prepared do you/did you feel for discharge from the neonatal unit?	Preparation for leaving unit

Did someone make you aware of the support that is available to you for when you do get home, for example, coming to A&E, seeing your GP, support from family?	Awareness of support available
Do you have any particular concerns for your baby once they are at home, having had surgery?	Concerns at home
Are there any additional concerns or practical problems caused by the pandemic?	Other concerns

Supplementary Table 2. An example of a summary template from our analysis
