# Supplementary material for: Learning from parental experience in a neonatal surgical unit: a qualitative service evaluation
Source: World J Pediatr Surg. 2023 Jul 10;6(3):e000596. doi: 10.1136/wjps-2023-000596 (PMC10335473; doi:10.1136/wjps-2023-000596)
Supplement: Supplementary data [file wjps-2023-000596supp003.pdf]

Before Admission

Knowledge of the baby's condition

Themes	Quotes	Actions	Service improvements
Difficulty processing antenatal diagnosis<br><br>The need for clear information in this period<br><br>Potential for unintended consequences of knowing there may be an abnormality<br><br>Importance of balanced antenatal discussion to managing expectations	*"Was told but didn't want to believe it"*<br><br>*"Conflicting information carried on throughout the whole antenatal period... If you are already anxious, nervous and have no idea what is going to happen, it is frustrating."*<br><br>*"It is really upsetting to talk about it to be honest. I don't think I have processed it myself yet."*	Feedback to staff on real world positive and negative effect of antenatal counselling<br><br>Need updated parent information leaflets	Monthly fetal medicine MDT clinic- includes neonatologist, obstetrician, surgical nurse, midwife and paediatric surgeon<br><br>Fetal medicine clinic reviewed 6 monthly<br><br>Antenatal counselling- includes support information- paediatric immediate life support, practical advice<br><br>Close links with family care team on NNU- meet parents, orientate to the ward, answer practical questions<br><br>Video tour of NNU now available
The value of trust in staff by parents who need confidence in professionals	*"Felt safe already, staff brilliant"*<br><br>*"I know someone from my hospital that was transferred here with their heart the wrong way round; so I know it is a good hospital"*	Feedback to staff and the potential to roll out feedback network-wide	Family care team<br><br>Poster about good communication tips for staff- considering parent feedback
Confusion when some congenital abnormalities were not detected on antenatal scans	*"We thought if anything would have been wrong, it would have shown on the amniocentesis"*<br><br>*"overwhelming at first…'felt hysterical at first but calmed down"*	Need to conduct work to determine what this group of families need to know in the immediate, short term and long-term<br><br>Consider postnatal meeting between foetal medicine and families if further explanation needed. This could include review of antenatal scans and review of performance to serve as audit	Neonatology team can link to obstetric team to meet parents for de-brief if required

Parents' general worries			
**Themes**	**Quotes**	**Actions**	**Service improvements**
Parents frightened about first going onto the unit<br><br>Parents worried about the wellbeing of their other children	*"petrified"*<br><br>*"didn't know what was going to happen"*	Video tour to show the rooms and incubator<br><br>'Supersibs' and sibling support packs potentially useful	Video tour of unit now done and being updated. This is shared antenatally<br><br>Neonatal operational delivery network (ODN) work stream to include videos for other units<br><br>Our neonatal unit podcast "Unexpected Beginnings- The Neonatal Unit". Hosted by veteran neonatal parents going through key aspects of being a parent on the neonatal unit to provide support for other parents, including an episode on 'Juggling Siblings'
COVID-19 specific issues			
**Themes**	**Quotes**	**Actions**	**Service improvements**
Difficulty of antenatal care with COVID-19 restrictions. It was difficult for the mother to attend scans and clinics alone, and difficult for the father to be excluded	*"I had to do all the scans on my own which I found really upsetting"*<br><br>*"It's like being separate families as being split up so often!"*<br><br>*"It was too much information to take in on your own"*<br><br>*"Husband felt left out and couldn't do anything"*	Consider video consultations as a standard offer when a parent is unable to attend because of COVID<br><br>Consider partner in terms of ongoing bonding and long- term well-being of the child as well as parents	Regular communication and letters given to all parents in relation to any IPC issues<br><br>Families kept updated
Parental uncertainty about risks of COVID-19 for baby<br><br>Conflicting information about COVID-19 protocols	*"scared that [baby] would get COVID-19 and she is obviously very vulnerable"*<br><br>*"The rules kept on changing all the time. You would ask someone questions about our appointments regarding COVID-19 regulations, and nobody really knew the answer"*	Collaborate with local, regional and national teams<br><br>Regional Q&A or FAQ as an efficient mechanism of information dissemination	Parental feedback on COVID-19 concerns and communication disseminated to all teams involved in neonatal care<br><br>Presented regionally and nationally also

Preparation for Transfer			
**Themes**	**Quotes**	**Actions**	**Service improvements**
Parents found transfer very stressful and found it hard not to be allowed to travel with baby	*"The transfer from one unit to another unit was the worst part of it all. It took a long time for the transfer to happen, but the people were lovely"*	Feedback to local transport network and involve them	Members of research team are actively involved in Neonatal Operational Delivery Network (ODN) co-production work streams to include project outcomes in all work eg<br>-transfer of surgical neonate information resources about all neonatal units in the region<br>-align practiced regionally to reduce the variability of care across the regional NNU<br><br>Working with the neonatal ODN and local neonatal transport service to help families access information and resources about transfer and the different type on neonatal units<br><br>Our regional neonatal transport team routinely collect feedback on care and transport experience with a quick response (QR) code on the incubators/beds. As a transfer involves care in multiple centres this inherently collects feedback on the differences between centres. Our unit is looking into using bedside QR codes as a tool to collect timely feedback.

Supplementary Table 3. Before admission

Admission to Hospital

Initial phase of care

Themes	Quotes	Actions	Service Improvements
Appreciation for welcoming team	*"to say it was a really rubbish situation it was really lovely, they were all great, understanding"*<br><br>*'felt like a family'*	Feedback to all staff, especially nursery nurses, the very positive comments	Fetal medicine MDT workstream commenced to support service improvements- 6 monthly review meetings<br><br>Veteran parent volunteers back to support families<br><br>Metric to ensure parents spoken to in first 24 hours<br><br>Psychology service now in place<br><br>We need to reintroduce 'super sibs'- volunteer service supporting siblings on the neonatal unit
The need more support around transitions and initial admission	*"I started crying at the cot of a baby that was not mine, because I thought that was [baby]. That is how little information I had upon admission"*<br><br>*"I think a midwife should have taken me to neonatal, that would have been very helpful; they could have then said 'this is [baby]'s mum, could someone please show her around"*	Consider an 'Induction' for new families as we do for staff. Perhaps this could be a role of the surgical liaison neonatologist<br><br>Consider video tours of the unit that are updated with any ward change with in future waves of the pandemic	Welcome posters on NNU- practical information<br><br>Family care team involvement- orientation to unit etc<br><br>Named neonatologists/Surgeons<br><br>Two neonatal consultants are now assigned as designated links to the surgical team<br><br>Communicated to midwife team to bring mother on 1st visit<br><br>Plan to develop photo books of the unit/equipment etc

			"Unexpected Beginnings- The Neonatal Unit" podcast episodes on 'what is a neonatal unit?' and 'meeting your baby for the first time'
During Admission			
**Themes**	**Quotes**	**Actions**	**Service improvements**
Parents valued individualised care<br><br>Physical and emotional challenges will exist for all new mothers following the birth; some will be particularly vulnerable<br><br>Language barriers were addressed well. Interpreters ere offered and available but they were not always very effective. Some families preferred faster, easier and less official means	*"The beads, Vcreate etc., white board were all very helpful. It was nice we could decorate the cot, it was all personal"*<br><br>*"young mums need a bit more support and a bit more explanation"*<br><br>*"I had had a C-section on a different ward and unable to go anywhere, while [baby] was going to theatre, I could not come to [baby], nobody was able to come and get me"*<br><br>*"Language barriers are professionally dealt with"*<br><br>*"Some interpreters difficult to understand"*<br><br>*" I would be happy with google translate sometimes"*	Tailor information and advice to the specific family. We need to be aware of specific groups and characteristics that may need tailored care. These could include; 'young' mums, family that conceived through IVF, those with language barriers, families with other health needs<br><br>Feedback on interpreting services and their use. Think how best to use interpreting services; Offer translation in different ways (person/app/google) for day to day work. Need to consider information governance risks phone applications	Simple measures were very appreciated and helpful<br><br>Badgernet videos regularly utilised<br><br>Events to promote with staff on special occasions eg Mother's Day, Fathers Day, Easter, birthdays etc<br><br>Staff utilising virtual interpreting services more often in addition to face-to-face: telephone, video, google translate

Operational issues			
**Themes**	**Quotes**	**Actions**	**Service improvements**
Importance of clear introductions and clear channels of communication	*“important to have one person you can talk to, one person you are happy with, you could go to talk to and trust”*<br><br>*‘big unit, moved rooms a lot so didn’t get to know many nurses well; Was told who surgeon was but did not meet until after surgery’*	Named nurses and named consultants needed (medical and surgical) and ensure the link between the neonatal and surgical teams is clear<br><br>Reminder to staff importance of introducing themselves and their role	Parents actively encouraged to attended ward round. Their attendance is recorded and audited across the service<br><br>Named consultants<br><br>Name boards at bed space for families to see<br><br>Joint neonatal and surgical ward rounds daily<br><br>Discussion with expert transport teams to develop feedback mechanisms so families can feedback on whole of healthcare journey
Building, environment, and practical issues			
**Themes**	***Quotes***	***Actions***	***Service improvements***
Appreciation of accommodation with the clean, spacious, private rooms nearby. This eased financial worries<br><br>Appreciation for WIFI<br><br>Travelling and parking a common source of stress as well as the indirect expense of the admission	*“a life-saver”*<br><br>*“it’s not a hotel but everything is perfect still”*<br><br>*“It is extremely expensive to have a baby admitted to LGI due to the costs around it”*<br><br>*“Got permit for free parking but there was still not always a parking space. Drove around a lot”*	Acknowledge the financial strain associated for these families<br><br>Inform planners of new building that the kitchen is very important, and parents need their own space within this. And note the importance of wifi to the Digital Leeds project mentioned by commissioners<br><br>No easy parking solution. Perhaps promote public transport and be clear with parents what the parking situation is like, so they know what to expect	Family care team and lead oversee all aspects of parental care & support<br><br>Family integrated care well established on NNU<br><br>Financial support from hospital charity for families<br><br>Some meal and refreshment provision. Ability to order parent food out of hours for families transferred in<br><br>Families have access to hospital parent accommodation/ facilities-free of charge. Designated parent facilities-kitchen, sitting/dining room

			Free parking permits available for families.<br>Some designated parking spaces protected for neonatal family use
Issues with building structure and lack of privacy	*"all parents could hear about our child and we knew about theirs"*<br><br>*"There was no natural light. I could not believe the room would be designed like this, and this was ridiculous to deprive the babies of this much light."*<br><br>*"The rooms are so loud, what with the building being this old, which is bad for the babies I believe."*	With the planned new build, we need a mix of single rooms and open bays, private space for families and more reclining chairs<br><br>Ensure appropriate access to screens/rooms/secure environment for expressing	New screens purchased to help provide more privacy in surgical newborns area. Screens have an 'under the sea' theme to match the room décor. The screens are wall mounted between each bed space<br><br>There is a designated breastfeeding room and parent consultation room already in place for additional privacy

Supplementary Table 4. Admission to hospital

Information and Support on the NNU

General

Themes	Quotes	Actions	Service Improvements
Information was clearly, presented, diagrams were useful<br><br>Parents distressed when they searched online to find information	*“The amount of information about the baby was enormous; Thanks to the quality of the team, I understand everything”*<br><br>*“It is difficult to understand; diagrams have solved all this; It is not the difficulty of the information that was upsetting, but the content and how rushed everything inevitably was”*<br><br>*“a bad move because you only ever remember the bad stuff from those websites”*	Encourage use of diagrams, have some standard diagrams of normal anatomy and some showing abnormalities we encounter what we can then draw on<br><br>Ensure good written and web resources for parents provided or signposted	Neonatal project work stream set up to help review and implement service improvements<br><br>Photo books planned to be developed- linking in with ODN co-production work streams<br><br>Surgical outreach signpost parents to resources/websites/charities<br><br>All parent information leaflets reviewed for quality of information and will be available shortly for families and staff
Parents felt the team were always honest, even if giving bad news	*“as much as I didn’t want to hear it, looking back I think well, I needed to hear it. As much as it’s a job for them, they’re doing the best possible thing which is being honest.”*<br><br>*“I didn't feel that they were keeping any secrets. That’s sometimes what you worry about, is there something they are not telling me.”*	Feedback to staff their candour is noticed and appreciated	Continue this care
Care felt personal	*“is almost like you have the same nurse for the whole time really that you are in hospital because they all know what is going on and it is great”*<br><br>*“to have a bit of a giggle amid all the seriousness felt very human”*<br><br>*“The family care team was absolutely amazing. Always present, every single day. ‘Do you need this’, ‘do you need that’. It was brilliant”*	Aim for consistency in staff over different shifts	Positive feedback to staff given to share feedback from families and to encourage continues good practice for supporting families<br><br>We need to review process for continuity of care from nursing team
Feeling of being overwhelmed by having an unwell baby	*“I basically said goodbye to him before surgery thinking the worst. I completely understand that they would paint the worst picture”*	Prepare parents as best as possible with explanations, photos and videos	Development of psychosocial MDT underway - links with unit safeguarding nurses

	"I never knew anything about neonatal medicine, so it was completely new"		
Some poor communication	*"We never got the amount of information about the stoma we wanted …Nobody came to sit down with us about that procedure before it happened"'*<br><br>*"Communication could have been better and promises should be kept: Excluded from room during precious visiting time because of procedures with other babies; procedures delayed and parents not updated as promised"*<br><br>*"Would like to have spoken to surgeons more, the surgeon actually doing operation we only caught by chance and we had about 30 seconds to speak to them"*	We need to work on improving communication. Specific areas highlighted that we need to be aware of include:<br><br>- Anomaly screen is particularly stressful – i.e., looking for other abnormalities when one has been found<br><br>- Less information is available at weekends<br><br>- Parents sometimes too tired to take things in – need things repeating<br><br>- Hard to hear staff when wearing a mask	Better joined up MDT working to help with communication, decision making and consistent care planning including:<br>- Joint neonatal and surgical ward rounds Mon-Friday<br>- Weekly multi-professional team meeting to have holistic oversight of progress ongoing care planning<br><br>Named consultants for each surgical neonate- neonatal and surgical - to lead with care, communication and decision making. Named consultants meets with family for updates<br><br>Name boards at cot-side so families know who named consultants are<br><br>Parents encouraged to attend ward rounds<br><br>Audit on parent presence on ward rounds<br><br>Nursing staff advocate for families and request meeting with surgeon/neonatologist if family request<br><br>Poster with details and picture of staff uniforms and job roles to help families understand different staff roles<br><br>Poster shared with staff with helpful 'top tips for good communication'. This was shared in Neonews (our weekly neonatal newsletter)

Conflicting information presents a large problem	*"I saw 4 consultants on different days, and everybody wanted to do something"*<br><br>*"If you change the plan every single day, how will you know if it was working or not?*<br><br>*"The teams did not really communicate well. They would tell us one thing and then 5 hours later they told us they were not going to do it anymore"*<br><br>*"It was a bit cross-wired, because someone had said he had had a good night, but then it turned out he had not had a good night and was sick a lot"*	Need to improve internal communication for consistency<br><br>If there are different opinions or the plan needs to change then we need to explain why	As previous
**Specific**			
Parents were aware of the counsellor but low uptake of service	"..might have been easier if it was in person. Talked to the staff instead"<br><br>*"the nurses' job is even harder now, where you have to be additional emotional support because the parents don't have it from anywhere else"*	Needs to be more visible and accessible<br><br>Nurses seen to be very supportive, but particularly important for families when isolated from other support	Psychology service now embedded on NNU<br><br>Hospital Chaplaincy team starting to facilitate weekly coffee morning to support families<br><br>Lots of resource posters with QR codes to help signpost families to support<br><br>Hospital charity funds available to apply for families needing financial support
Bereavement care	*"The stuff they did before X died and afterwards, it were never too much. Like we left some of his clothes here and they posted them out to us... I don't think I could have been as strong as I was if they weren't as strong as they were as well"*	Feedback to staff<br><br>Encourage ongoing training with butterfly trust	Funding for a bereavement nurse- not in post yet<br><br>Close links established with regional hospices<br><br>Bereavement clinical room available
Video links are helpful in reducing anxiety	*"a God-send and helped us through… For any parent, mum or dad, it is the best thing"*	Extend the utilisation of virtual platforms to support families	Bagdernet video diaries used frequently to connect families with their baby when not on the unit

Supplementary Table 5. Information and support on the neonatal unit

Impact of COVID

COVID-19 information

Themes	Quotes	Actions	Service Improvements
Parents generally felt well informed and accepted the rues<br><br>Frustration about rule changes	*"there were signs everywhere. I was told in advance when I had to get my covid test, when dad was supposed [to get his]"*<br><br>*"Don't really believe in Covid but accept restrictions"*<br><br>*"The rules kept on changing all the time… You would ask someone questions about our appointments regarding COVID-19 regulations, and nobody really knew the answer"*	Be transparent regarding uncertainty or when things have changed	Regular communication and letters given to all parents in relation to any infection prevention and control (IPC) issues

COVID-19 worries

Themes	Quotes	Actions	Service Improvements
The potential risk to the baby was a constant additional worry	*"Covid always at the back of the mind"*	Be mindful of the impact of covid and how worrying it is	The COVID-19 team at Leeds Children's Hospital provided written information<br><br>More psychological support available

What families thought went well

Themes	Quotes	Actions	Service Improvements
Feeling safe that the staff followed rules and visitors were not allowed	*"tested regularly - what more could they do - Really good, doing everything"*<br><br>*"I did not feel unsafe at any time. I have seen the staff cleaning their hands, changing their aprons, gloves etc"*<br><br>*"I think the pandemic made it kind of safer"*	If any further waves we should be reassured our measures made parents feel safe<br><br>Acknowledge previously unrecognised positive impact of the infection control rules eg some parents enjoy stricter visiting policies	More information about testing
Frustration that there were sometimes double standards – social distancing for parents	*"told off for not social distancing but then the staff were all round the work station – looks like work most of the time but not always! Didn't like that"*	Staff must follow rules	Written information was created which acknowledged that rules may change over the course of the pandemic. This recognised the

but staff congregate around station<br><br>Discrepancies between hospitals	*"It was just strange that the medical staff were not being swabbed"*<br>*"There did seem to be a little discrepancy regarding family visits"*<br><br>*"We were so happy that the rules in Leeds were more lenient towards that"*	Be open to people pointing out discrepancy and be able to explain or correct<br><br>Need consistency of rules between different wards and units or explanation for differences<br><br>Comparison with other centres needs feedback to network real time to identify useful differences	variability between centres and over time.<br><br>Written information also explained that beyond COVID-19 there are differences between hospitals, and it is not possible and not always appropriate for centres to be the same<br><br>Paediatric Grand Rounds provided a time to highlight any concerns
The restrictions caused stress. Parents worried about their other children not bonding with the baby and worried about the development of the baby who is only interacting with people hidden behind personal protective equipment	*"Sibling only sees on facetime/photo. Doesn't talk about baby"*<br><br>*"siblings didn't see twin before he died"*<br><br>*"these are Newborns the first few weeks of their lives are critical for bonding"*<br><br>*"they learn a lot from facial expressions"*<br><br>*"the only people who were touching her were medical staff. For 5 weeks we did not hold our baby, which made the bond we initially had disappear only people touching her were medical staff for interventions"*<br><br>*"It must be horrible for babies to just feel rubber and plastic all the time"*<br><br>*"the nurses got [sibling] a Build-a-Bear, a storybook, which was brilliant, and helped us massively. [Sibling] is 5 years old. He realises he has a brother, and is able to give him little presents, and keeps being interested in all the updates, but has never managed to see him in real life"*	Nurses very engaged in helping siblings bond – positive feedback for build a bear<br><br>Acknowledge impact on siblings and offer advice<br><br>In end-of-life cases we should reconsider sibling visiting. The siblings weren't able to visit a dying sibling, they had to wait until he had already died and was in a hospice. This seems very difficult and needs addressing	'Sibling packs' created to ensure siblings that were unable to visit had activities to involve them in care<br><br>Understanding of the importance of relatives visiting for end-of-life care led to more visiting being allowed in end-of-life care as the pandemic progressed

Supplementary Table 6. Impact of COVID-19

Discharge

Preparedness for leaving the unit

Theme	Quotes	Action	Service Improvements
Parents generally felt well supported in preparation for discharge<br><br>Can be stressful if discharge delayed<br><br>Importance of support from clinical nurse specialists, community team, and outreach nurses<br><br>Some realised with hindsight that they were not as well prepared psychologically as they thought	*"The LGI gave us all the information we needed"*<br><br>*"The outreach nurses have been amazing"*<br><br>*"Bit of a shock to the system….but nothing more could've been done - getting psyched up"*<br><br>*"I don't think I was prepared in a way. I think I thought I was."*<br><br>*"We were desperate to leave hospital"*	Ensure family utilise Family Integrated Care documentation re discharge planning<br><br>Consider wider family in discharge planning- utilise virtual forums for training/information<br><br>Need clear discharge criteria and communicate this to family. If discharge is 'goal based' then families will be perhaps less focussed on an agreed time that may then change<br><br>Reality of discharge different than expected – we need to work on this transition and managing expectations	Multi-disciplinary discharge meetings arranged for more complex infants<br><br>Family integrated care imbedded in the ethos of care delivery.<br><br>Discharge planning document utilised<br><br>Weekly 'discharge hudddle' to discuss patient flow and what needs to be completed and any family needs, teaching, information etc plus weekly MDT ward round to highlight discharge needs.<br><br>Feedback to the neonatal surgical outreach team the positive feedback on their involvement<br><br>Extra BLS training sessions for staff so more staff able to support parental training and reduce delays on discharge<br><br>Implementation of criteria led discharge- helps manage parental expectations and reduce delays on day of discharge
Managing bereavement of a newborn	*"There is nothing I can imagine that could have helped us more than was already done for us after the baby's passing"*	Continuity of care important for end-of-life care	

Adjusting to life at home			
**Theme**	**Quotes**	**Action**	**Service improvements**
Good follow up support provided<br><br>Re-admittance can be unexpected and difficult - some happy if needed to come back in, some not as prepared as they thought<br><br>Many parents had built up a high level of trust in the hospital team, and sometimes worried about a perceived lack of expertise in their local hospital/ health visitor when home	*"in safe hands"*<br><br>*"We felt prepared to go home. We just had not anticipated that he would have to be readmitted to hospital. I do not think anyone could have known"*<br><br>*"Even now that we have been discharged, we are always assured to have contact with the ward in case something goes wrong or for more questions"*	Continue to signpost clear communication channels for families at home<br><br>Acknowledge that readmissions emotionally difficult - need preparation<br><br>Professional conversations with family needed to reduce lack of trust in other organisations	Current service review of Neonatal surgical outreach provision and what service needs are<br><br>Aim to improve parent contacts and availability for specialist discharge planning and education<br><br>Outreach nurses go to district general hospitals (DGHs) to facilitate earlier repatriation and joined up care with DGH and tertiary centre.<br><br>DGH team invited to MDT virtually if there is a surgical neonate returned to local centre
The difficulty of adjusting to life at home<br><br>Feeling isolated at home on discharge<br><br>Appreciation for community teams	*"We had to get used to life out of hospital. All the alarms, beeping and those noises"*<br><br>*"It is a strange feeling to have your baby at home after long invasive support, which is why still having access in the community is essential"*<br><br>*"I just have to be more careful and really watch my baby for any signs"*<br><br>*"quiet but struggling"*<br><br>*"There's always those things in the back of your mind and you don't want to sound an idiot to the doctors asking what if, what if, what if?"*<br><br>*"We haven't really seen anyone since we came home. … It has just been us in our own little bubble. It is difficult some days"*	Feedback to community teams<br><br>Make time to answer all questions prior to discharge<br><br>Need to signpost sources of support for parents including outreach, GP, health visitors, mental health support	Feedback to community team<br><br>"Unexpected Beginnings- The Neonatal Unit" podcast episode 'Going home' explores issues around getting reading to go home, adapting to life away from the neonatal unit, managing the associated anxiety and the role of the transitional unit in the process<br><br>Good coordination of discharge and communication facilitates. Needs some review and improvement

Supplementary Table 7. Discharge from hospital
